# Evaluating a semi‐nested PCR to support histopathology reports of fungal rhinosinusitis in formalin‐fixed paraffin‐embedded tissue samples

**DOI:** 10.1002/jcla.24209

**Published:** 2022-01-08

**Authors:** Mohammad Javad Ashraf, Mohammad Kord, Hamid Morovati, Saham Ansari, Golsa Shekarkhar, Hamid Badali, Kayvan Pakshir, Forough Shamsizadeh, Bijan Khademi, Mahmood Shishegar, Kazem Ahmadikia, Kamiar Zomorodian

**Affiliations:** ^1^ Department of Pathology School of Medicine Shiraz University of Medical Sciences Shiraz Iran; ^2^ Department of Parasitology and Mycology School of Medicine Shiraz University of Medical Sciences Shiraz Iran; ^3^ Department of Medical Parasitology and Mycology School of Medicine Shahid Beheshti University of Medical Sciences Tehran Iran; ^4^ Fungus Testing Laboratory Department of Pathology and Laboratory Medicine University of Texas Health Science Center at San Antonio San Antonio Texas USA; ^5^ Invasive Fungi Research Center Communicable Diseases Institute Mazandaran University of Medical Sciences Sari Iran; ^6^ Basic Sciences in Infectious Diseases Research Center Shiraz University of Medical Sciences Shiraz Iran; ^7^ Research Center of Otolaryngology Head and Neck Surgery Shiraz University of Medical Sciences Shiraz Iran; ^8^ Department of Otolaryngology School of Medicine Shiraz University of Medical Sciences Shiraz Iran; ^9^ Department of Medical Parasitology and Mycology School of Public Health Tehran University of Medical Sciences Tehran Iran

**Keywords:** formalin‐fixed paraffin‐embedded tissue, fungal rhinosinusitis, histopathology, semi‐nested PCR, sequencing

## Abstract

**Background:**

Fungal rhinosinusitis (FRS) encompasses a various spectrum of diseases. Histopathology is the “reference method” for diagnosing FRS, but it cannot determine the genus and species. Moreover, in more than 50% of the histopathologically proven cases, the culture elicited no reliable results. This study was an attempt to evaluate the diagnostic efficiency of semi‐nested polymerase chain reaction (PCR) from formalin‐fixed paraffin‐embedded (FFPE) functional endoscopic sinus surgery (FESS) in FRS patients.

**Methods:**

One hundred ten specimens were subjected to DNA extraction and histopathology examination. The amplification of the β‐globin gene by conventional PCR was used to confirm the quality of extracted DNA. The semi‐nested PCR was performed using ITS1, ITS2, and ITS4 primers during two steps. Sequencing the internal transcribed spacer region (ITS1‐5.8S‐ITS2) to identify causative agents was performed on PCR products.

**Results:**

Sixty‐four out of 110 samples were positive by histopathology evidence, of which 56 samples (87.5%) were positive by PCR. Out of 46 negative samples by histopathological methods, five samples (10.9%) yielded positive results by PCR. Sensitivity, specificity, positive predictive value, and negative predictive value of the semi‐nested PCR method were reported 87.5%, 89.2%, 92.7%, and 85.2%, respectively. The kappa factor between PCR and histopathological methods was 0.76, indicating substantial agreements between these two tests.

**Conclusion:**

Due to the acceptable sensitivity and specificity of the present method, it might be used to diagnose fungal sinusitis infections along with microscopic techniques. This method is recommended to confirm the diagnose of suspected fungal sinusitis with negative histopathology results.

## Graphical abstract

Following clinical susception, for the fungal rhinosinusitis (FRS), the samples were collected from the rhino‐nasal cavity of 110 patients and were stained by hematoxylin and eosin (H&E), periodic acid‐Schiff (PAS) stains. For the molecular assay, 110 specimens were subjected to DNA extraction and histopathology examination. The amplification of the β‐globin gene by conventional PCR was used to confirm the quality of extracted DNA. The semi‐nested PCR was performed using ITS1, ITS2, and ITS4 primers during two steps. Sequencing the internal transcribed spacer region (ITS1‐5.8S‐ITS2) to identify causative agents was performed on PCR products. Sixty‐four out of 110 samples were positive by histopathology evidence, of which 56 samples (87.5%) were positive by PCR. Out of 46 negative samples by histopathological methods, five samples (10.9%) yielded positive results by PCR. Sensitivity, specificity, positive predictive value, and negative predictive value of the semi‐nested PCR method were reported 87.5%, 89.2%, 92.7%, and 85.2%, respectively. The kappa factor between PCR and histopathological methods was 0.76, indicating substantial agreements between these two tests.

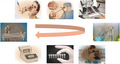

## 1 BACKGROUND

The inflammation of paranasal sinus mucosa is called sinusitis, or rhinosinusitis,^1^ which commonly affects approximately 4.5%–12% of the North American and European populations and 20% of the world population.^2^, ^3^, ^4^ Depending on risk factors, colonization of fungal spores in sinonasal cavities triggers immunopathological consequences, fungal sinusitis, or more accurately, fungal rhinosinusitis (FRS).^5^ FRS is being reported with increasing frequency worldwide.^5^ An expansion in the incidence rate of FRS is a consequence of global escalation in the immunocompromised population.^6^ However, the situation becomes more complicated with the significant increase in FRS reported in immunocompetent hosts without predisposing factors.^7^

Fungal rhinosinusitis includes a wide range of the clinical spectrum that encompasses the mild form of superficial colonization and allergic manifestations to life‐threatening invasive disease.^5^ FRS is divided into two categories from a histopathological aspect, that is, invasive and noninvasive infection, depending on tissue invasion of the mucosal layer. The invasive infection includes acute invasive (fulminant) FRS, granulomatous invasive FRS, and chronic invasive FRS, while noninvasive diseases include localized colonization, fungal ball, and fungus‐related eosinophilic FRS or allergic fungal rhinosinusitis (AFRS).^5^, ^8^, ^9^

Diagnosis is always a significant challenge in the management of FRS. In addition to clinical manifestations and radiological data, which are nonspecific, available laboratory methods are direct microscopic examination by potassium hydroxide (KOH), histopathology by Gomori methenamine silver (GMS) and hematoxylin and eosin (H&E), culture, antigen/antibody testing (galactomannan and *Alternaria* antigen tests), and molecular methods.^10^, ^11^, ^12^, ^13^, ^14^

In FRS cases, the culture of obtained specimens may yield false‐positive or false‐negative results, respectively, because of environmental contamination during sampling and loss of viability due to improper transfer and storage conditions.^15^

The histopathological method is considered a reference method for diagnosing FRS and is essential in categorizing this infection.^11^ However, previous researches indicate that approximately 3.5%–36% of FFPE histopathological examinations may produce false‐negative results.^16^, ^17^, ^18^

On the contrary, preparing and staining tissue specimens is a time‐consuming procedure that requires a trained individual with extensive knowledge. *Mucorales* are responsible for about 45%–75% of FRS^19^, ^20^, ^21^, ^22^ and have a fast invasion with significant morbidity and mortality in the event of a compromised immune system. Thus, early detection of this infection is critical. As a result, a supplementary reliable diagnostic technique on the same sample is required to support and corroborate the histopathological findings. Because the specimen obtained through endoscopy‐guided biopsy is mainly used to make histopathological diagnoses, employing a reliable molecular technique for direct detection of FRS in the residual FFPE samples may enhance diagnosis. Hence in this study, a semi‐nested PCR in FFPE samples was used to detect FRS compared with the histopathology examination.

## 2 MATERIALS AND METHODS

### 2.1 Samples and patients

The functional endoscopic sinus surgery (FESS) samples were collected from 110 patients (64 positive FRS and 46 non‐FRS) in the prospective cross‐sectional study. The status of patients was proved by clinical signs and symptoms and computed tomography (CT) scan. Demographic data of the patients, including age, sex, type of operation, site of infection, background diseases, and final pathology report, were documented (Table 1). The paraffin blocks were prepared for the cutting process by microtome. During this process, ten slices were randomly cut with a thickness of 5 μm from each of the PEBs. The samples were put into microtubes to further molecular investigations.

**TABLE 1:** Demographic data of the patients and the results of molecular assays

Patient no	Sex	Age	Risk factors	Pathology report	Outcome	PCR β‐globin/ITS	Sequence
1	F	41	ND	Chronic FRS	Survived	+/+	*A. flavus*
2	F	50	ND	Chronic FRS	Survived	+/+	*C. albicans*
3	F	40	ND	Chronic FRS	Survived	+/+	*A. flavus*
4	F	52	Leukemia	Mucormycosis	Died	+/+	‐
5	F	42	Diabetic	Mucormycosis	Survived	+/+	*R. oryzae*
6	F	50	Leukemia	Acute FRS	Died	+/+	*A. flavus*
7	M	55	ND	Mucormycosis	Died	+/−	‐
8	M	2	Leukemia	Chronic FRS	Survived	+/+	*A. flavus*
9	F	63	Leukemia	Mucormycosis	Survived	+/+	‐
10	M	21	Allergy	Chronic FRS	Survived	+/+	*Cr. ozbekistanensis*
11	M	51	ND	Chronic FRS	Survived	+/+	*A. flavus*
12	M	26	Leukemia‐Allergy	Mucormycosis	Survived	+/−	‐
13	M	58	ND	Chronic FRS	Survived	+/+	*A. flavus*
14	F	47	ND	Chronic FRS	Survived	+/+	*C. albicans*
15	M	32	Allergy	Chronic FRS	Survived	+/+	*A. fumigatus*
16	M	26	ND	Chronic FRS	Survived	+/+	‐
17	F	42	ND	Mucormycosis	Died	+/+	*R. oryzae*
18	M	43	ND	Chronic FRS	Survived	+/+	‐
19	M	20	Diabetic	Mucormycosis	Survived	+/+	‐
20	F	49	ND	Mucormycosis	Survived	+/+	*R. microsporus*
21	F	42	Diabetic	Acute FRS	Survived	+/+	*A. oryzae*
22	M	25	Leukemia	Mucormycosis	Survived	+/+	‐
23	M	36	Diabetic	Mucormycosis	Died	+/+	‐
24	F	44	ND	Mucormycosis	Died	+/+	*L. corymbifera*
25	F	53	ND	Mucormycosis	Survived	+/+	‐
26	F	35	Leukemia	Mucormycosis	Died	+/+	‐
27	F	32	ND	Chronic FRS	Survived	+/+	*A. alternate*
28	M	26	ND	Chronic FRS	Survived	+/+	*A. parasiticus*
29	F	70	Leukemia	Chronic FRS	Survived	+/+	*A. flavus*
30	M	53	Leukemia	Mucormycosis	Survived	+/+	‐
31	F	25	Leukemia	Mucormycosis	Died	+/+	‐
32	M	65	ND	Mucormycosis	Survived	+/−	‐
33	F	20	Leukemia	Mucormycosis	Died	+/+	‐
34	F	52	Addiction	Chronic FRS	Died	+/+	*A. flavus*
35	M	55	ND	Mucormycosis	Survived	+/+	‐
36	M	55	Diabetic	Mucormycosis	Survived	+/+	*R. oryzae*
37	F	59	Leukemia	Mucormycosis	Died	+/+	*R. oryzae*
38	F	59	Leukemia	Mucormycosis	Died	+/+	*R*. *oryzae* ‐
39	M	34	Polyp	Acute and chronic FRS	Survived	+/+	*A. flavus*
40	F	37	Leukemia	Mucormycosis	Died	+/+	‐
41	F	64	Autoimmune disease	Mucormycosis	Survived	+/−	*R. oryzae*
42	F	61	ND	Chronic FRS	Survived	+/+	‐
43	F	6	Leukemia	Mucormycosis	Died	+/+	‐
44	M	52	Transplantation	Mucormycosis	Died	+/+	*R. oryzae*
45	M	50	Diabetic‐Transplantation	Mucormycosis	Died	+/+	*R. oryzae*
46	M	51	Transplantation	Mucormycosis	Died	+/−	‐
47	F	55	Diabetic‐Transplantation	Mucormycosis	Died	+/+	*Saksenaea vasiformis*
48	M	20	ND	Mucormycosis	Died	+/+	‐
49	F	53	ND	Mucormycosis	Survived	+/+	*L. corymbifera*
50	F	51	Diabetic‐Leukemia	Mucormycosis	Survived	+/+	‐
51	F	52	ND	Chronic FRS	Survived	+/+	*A. flavus*
52	F	34	Diabetic‐Leukemia	Mucormycosis	Survived	+/+	‐
53	M	56	Diabetic	Mucormycosis	Died	+/+	‐
54	M	30	Leukemia	Mucormycosis	Survived	+/+	*R. oryzae*
55	F	62	Diabetic	Mucormycosis	Died	+/−	‐
56	F	49	Diabetic	Chronic FRS	Survived	+/+	*C. albicans*
57	M	6	Leukemia	Mucormycosis	Survived	+/+	‐
58	F	46	Diabetic	Chronic FRS	Survived	+/+	*A. flavus*
59	F	22	Leukemia	Mucormycosis	Died	+/+	‐
60	M	51	Leukemia	Mucormycosis	Survived	+/+	*R. oryzae*
61	M	33	Diabetic	Chronic FRS	Survived	+/+	*A. flavus*
62	F	47	Leukemia	Mucormycosis	Died	+/−	‐
63	F	4	Leukemia	Mucormycosis	Survived	+/+	*L. corymbifera*
64	M	9	Diabetic	Mucormycosis	Died	+/−	‐

### 2.2 Deparaffinization process

To reduce the contamination of samples during this process, it recommended the sterilization of microtome and other instruments using benzene and 2 M HCl rinsed with sterile water (www. leicabiosystems.com). The 1000 µL of xylene was added to microtube containing 5 µm of a sample, which was then incubated in 56°C on a heating block for 15 min at room temperature and section subsequently centrifuged at 10,000 × *g* for 2 min. The supernatant was removed, and 1000 µl of absolute ethanol was added and followed by centrifugation at 10,000 × *g* for 3 min. The previous stage was repeated three times, and then, the tubes were incubated at 37°C on a heating block until the total evaporation of the ethanol.^23^

### 2.3 Histopathological assay

The FFPE‐FESS samples were stained by hematoxylin and eosin (H&E), periodic acid‐Schiff (PAS) stains. The staining processes were performed according to the protocols for FFPE sample staining.^24^

### 2.4 DNA extraction

DNA was extracted as previously described. Briley, 100 μl lysis buffer, 180 µl of ATL buffer, and 20 µl of proteinase K were added to the tube samples. After overnight incubation at 56°C, the tubes were washed via normal saline. To complete the lysis process, tubes were heated in boiling water for 5 min. The tubes were incubated in boiling water and liquid nitrogen for 1 and 2 min, respectively. This step was repeated several times. Finally, they reached room temperature. As previously described, DNA extraction was completed by QIAamp DNA extraction from tissue mini kit (Qiagen, Hilden, Germany).^25^ This process is based on the binding of the DNA to silica columns.

### 2.5 PCR assay

To evaluate the quality of the extracted DNA, human β‐globin gene fragments amplificated by the PCR method^26^, ^27^ and KM29/PCO4 primers. PCR process was performed in a total volume of 25 μl. So that, the master mix containing 1mM MgCl_2_, 200 μmol/L deoxyribonucleotide triphosphates solution (dNTP)s, 1X reaction buffer 10×, 1 U Taq DNA polymerase (total of Cinna Gene, Iran), and 1 μl (10 picomols) of forward and reverse primers (Table 2). PCR conditions were as follows: denaturation phase 1 cycle at 94℃ for 10 min followed by 35 cycles of 94℃ for 45 s, 58℃ for 45 s and 72℃ for 5 min, and a final extension at 72℃ for 7 min. Amplicon quality and concentrations were estimated on the agarose gel and analyzed by the Gel Doc XR system (Bio‐Rad, USA). The smart Ladder (Eurogentec, Seraing‐Belgium) was used as the size and concentration marker.

**TABLE 2:** Primers used for internal control and detection of fungal DNA by semi‐nested PCR

Primers	5′–3′ sequence	References
β‐Globin (F:KM29)	GGT TGG CCA ATC TAC TCC CAG G	
β‐Globin (R:PCO4)	CAA CTT CAT CCA CGT TCA CC	
ITS1 (F)	TCC GTA GGT GAA CCT GCG G	
ITS2 (R)	GCT GCG TTC TTC ATC GAT GC	
ITS4 (R)	TCC TCC GCT TAT TGA TAT GC	

### 2.6 Semi‐nested PCR assay

The universal fungal ITS region (ITS1‐5.8S‐ITS2) was targeted for evaluation by semi‐nested PCR. The first PCR was performed using ITS1 (forward) and ITS4 (reverse) primers (Table 2). The total volume was 50 µL encompasses 45 µL of reaction mixture containing 1mM MgCl_2_, 1x of PCR buffer 10×, 0.1 mM each deoxynucleotide triphosphate (dNTP), 0.5 pmols/µl of each primer, 1 U of Taq DNA polymerase, and 5 µl of nucleic acid extract. In the second PCR step, the ITS1 (forward) and ITS2 (reverse) regions were amplified within the 3 µl of diluted (1/100) product of the first PCR step. The product of the first step was run into the second PCR to amplify ITS1 (forward) and ITS2 (reverse) regions in the same total volume (Table 2). PCR conditions for the first step were as follows: 10 min of initial denaturation at 94°C, 35 cycles of 96°C for 45 s, 58°C for 45s, and 72°C for 1 min, and a 5 min final extension at 72°C. Also, PCR conditions for the second step were as follows: 5 min of initial denaturation at 94°C, 32 cycles of 94°C for 30 s, 60°C for 30 s, and 72°C for 45 s, and 5 min final extension at 72°C.^28^ Both steps of the semi‐nested PCR encompassed 10–20 samples included a positive control containing 0.5 ng of purified DNA of one of the fungal isolates and at least two blanks with reagents only. Product quality and concentrations were estimated on the agarose gel and analyzed by the Gel Doc XR system (Bio‐Rad, USA). Smart Ladder (Eurogentec, Seraing, Belgium) was used as the size and concentration marker.

### 2.7 Sequencing

To further confirm the results, PCR products were sent to sequencing using referenced primers was performed. The obtained sequences were searched using the NBLAST algorithm (https://blast.ncbi.nlm.nih.gov/Blast.cgi), and the identity of each strain was assigned accordingly.

### 2.8 Data statistics

Data were analyzed using SPSS software version 24. Briefly, descriptive data were presented as mean, standard deviation, percentages, and charts. The chi‐squared and Fisher's exact tests were used to compare qualitative variables between the two groups. The Student *t* test was used to compare quantitative variables between the two groups. Also, the agreement between the two diagnostic methods was calculated by the Kappa test; interpretation of Kappa was based on Viera et al.^29^ The p‐value less than 0.05 (*p *< 0.05) was considered statistically significant.

## 3 RESULTS

### 3.1 Patients and samples

One hundred ten FFPE samples were obtained from 2018 to 2020. The patient's ages were ranged from 2 to 82 years old (mean age: 40.2 years old), with 50 being male (45.5%). Table 1 contains summarized demographic data for the patients. The majority of FRS patients had predisposing conditions, which included leukemia (20:64, 31.23%), diabetes (11:64, 17.11%), transplantation (4:64, 6.25%), allergies (3:64, 4.7%), polyp, autoimmune disorders, and drug addiction (1:64, 1.56%).

### 3.2 Histopathological examinations

**FIGURE 1:**
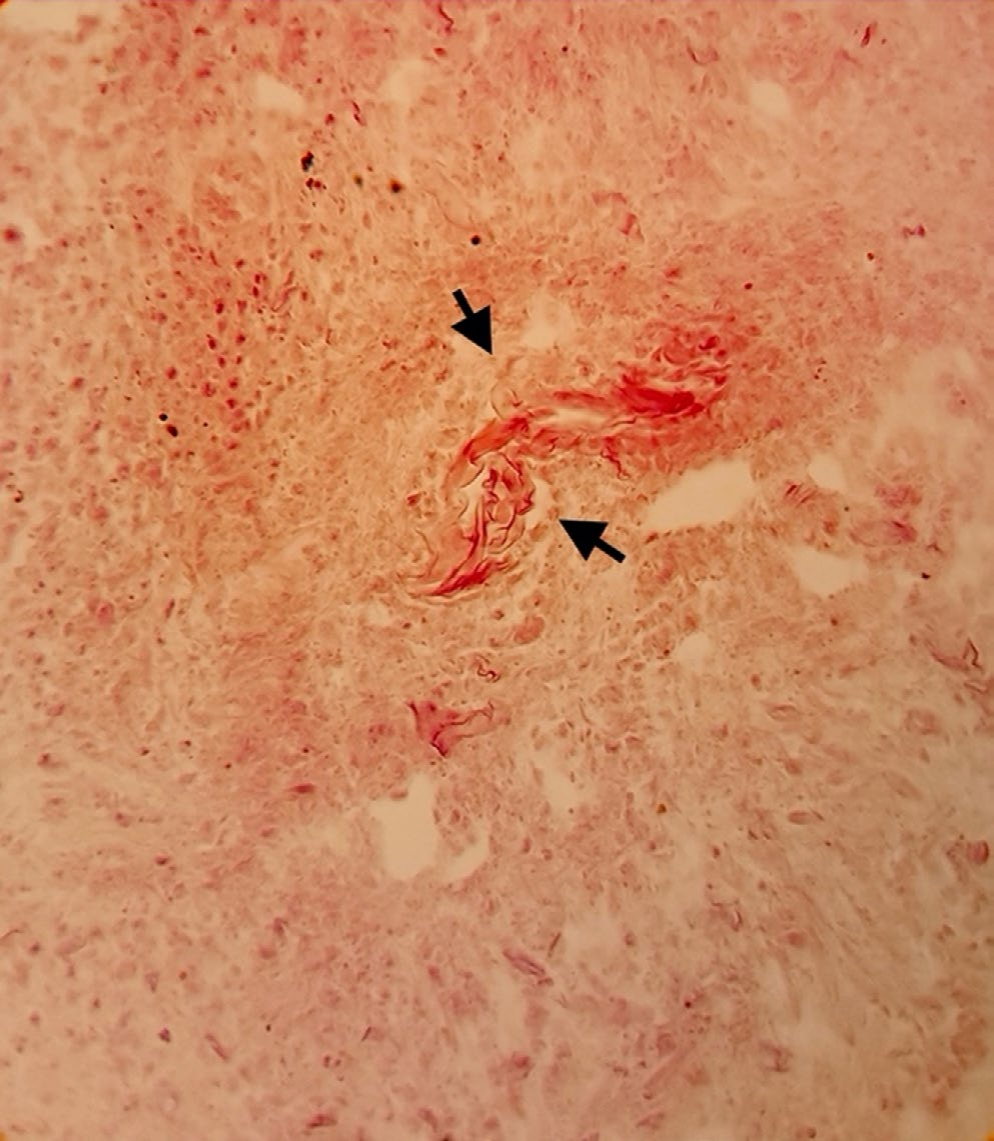
Mucor hyphae in a necrotic background of sinusal tissue, H&E stain (×400)

**FIGURE 2:**
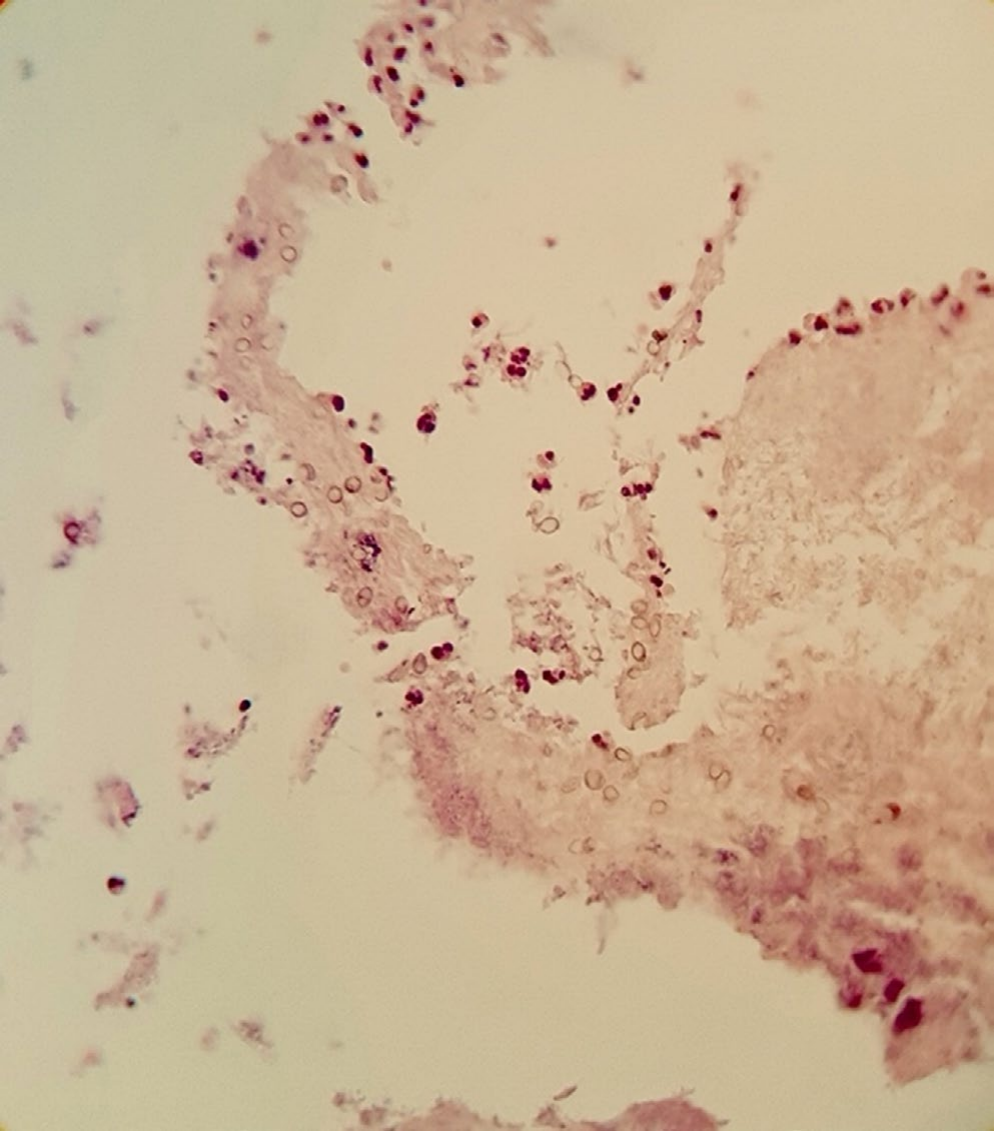
Fungal spores in the sinusal tissue of chronic sinusitis, H&E stain (×400)

**FIGURE 3:**
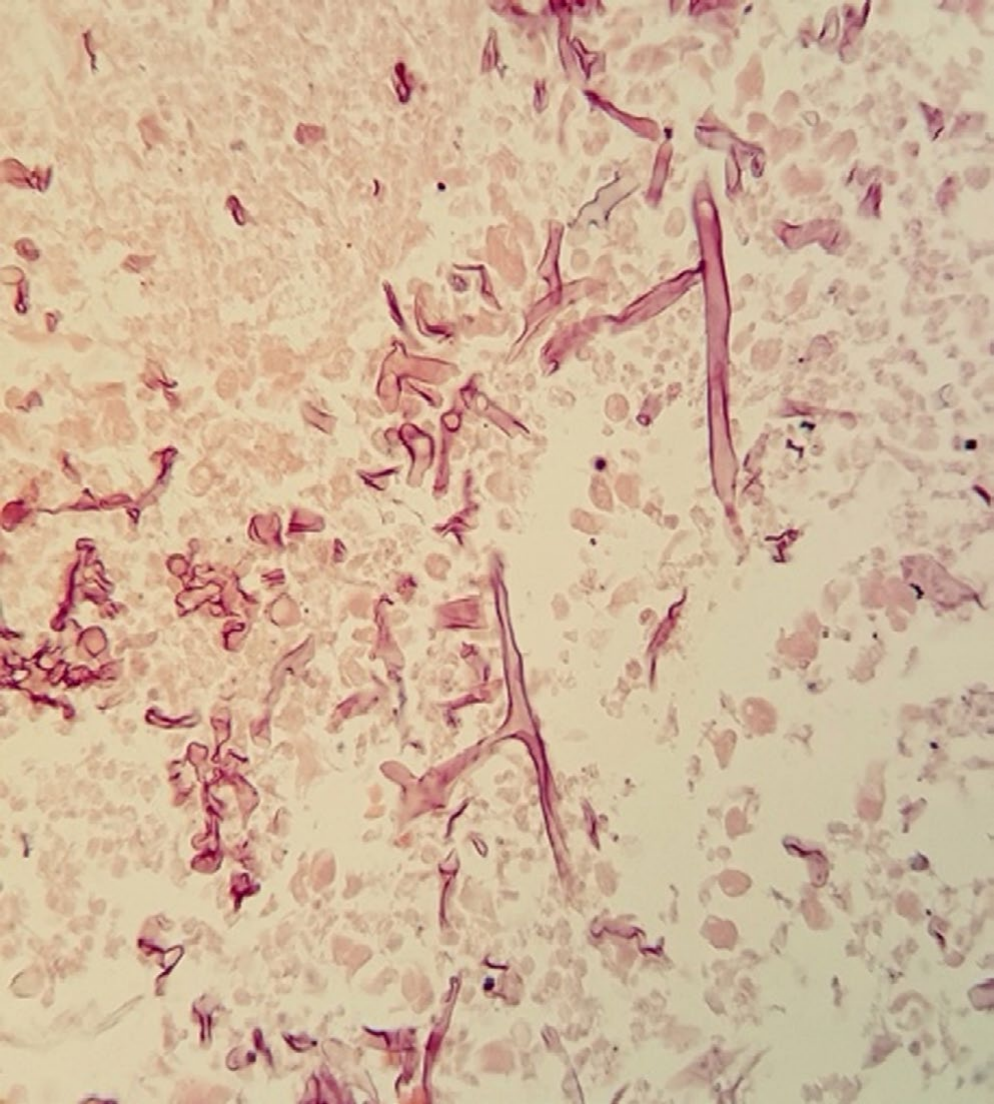
Non‐septate hyphae in the sinusal tissue, H&E stain (×400)

Fungal rhinosinusitis was found in 64 of the samples tested. Ribbon‐like non‐septate or slightly septate hyaline mycelium was found in 64% (41 of 64) of them, suggesting mucormycosis (Figures 1, 2, 3). Furthermore, pathology data revealed that 31% (20 of 64) of FRS cases were chronic, 3.12% (2 of 64) were acute, and 1.56% (1 of 64) were acute/chronic FRS (Table 1). As controls, 46 non‐FRS samples were included in the study.

### 3.3 Molecular assay

**FIGURE 4:**
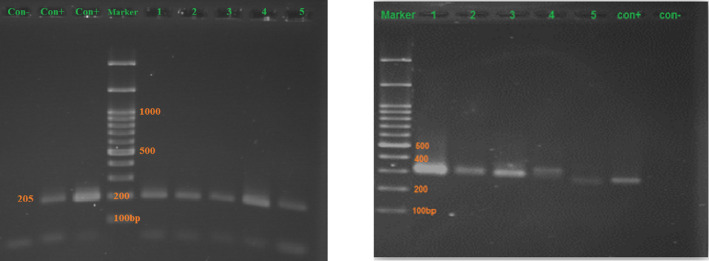
Gel electrophoresis primary PCR via β‐globin (left) and semi‐nested PCR for ITS region (right)

As shown in Table 3, 56 of the 64 (87.5%) histologically proven specimens were positive for PCR (as shown in Figure 4), while eight samples (12.5%) were negative. Out of 46 negative histopathological samples, 41 (89.1%) remained negative for PCR, while 5 (10.9%) were positive. Furthermore, the semi‐nested PCR method detected 100% (23/23) of chronic, acute, and acute/chronic FRS cases and 80.5% (33/41) of mucormycosis cases. Semi‐nested PCR had 87.5% sensitivity, 89.1% specificity, 92.7% positive predictive value, and 85.2% negative predictive value, respectively. The kappa value between these two tests was 0.76, indicating substantial agreement between these two tests. Thirty‐five PCR products were successfully identified from the 56 samples sequenced. This study's 35 sequences were deposited in the GenBank database (accession numbers from MZ333236 to MZ333270 with persistent accessible links from MZ333236 to MZ333270, respectively). Eventually, *Aspergillus flavus* (12/35), *Rhizopus oryzae* (10/35), *Lichtheimia corymbifra* (3/35), *Candida albicans* (3/35), *Aspergillus oryzae* (1/35), *A*. *parasiticus* (1/35), *A*. *fumigatus* (1/35), *Saksenaea vasiformis* (1/35), *Rhizopus microsporus* (1/35), *Alternaria alternata* (1/35), *and Cryptococcus uzbekistaniensis* (1/35) were identified by sequencing of the ITS region.

**TABLE 3:** Comparing the results of histopathology and PCR assay

Pathology report	PCR result	Number (%)
FRS (*n* = 64)	Positive	56 (87.5)
	Negative	8 (12.5)
Non‐FRS (*n* = 46)	Positive	5 (10.9)
	Negative	41 (89.1)
Sensitivity	87.5%	
Specificity	89.2%	
Positive predictive value	91.8%	
Negative predictive value	83.6%	

## 4 DISCUSSION

Several affect the outcome of the PCR test in FFPE tissue samples. The DNA extraction method, the inclusion of a suitable housekeeping gene, the PCR method (i.e., panfungal, specific, nested, semi‐nested, multiplex, and real time), the target gene(s) primers, the amplicon length, the thickness of the FFPE cut, the specimen storage time, and contamination during sample preparation are all factors to consider.^30^

This study found that a semi‐nested PCR test targeting the ITS1‐5.8s‐ITS2 (ITS1‐2) region of 110 FFPE‐FESS samples had a sensitivity of 87.5% and a specificity of 89.1%. Here, the PCR results were positive for 56 of the 64 histopathology positive samples; furthermore, the kappa factor between these two methods was 0.76, indicating substantial agreement between these two tests. Moreover, it was observed that all cases of negative PCR were associated with mucormycosis. Bialek et al. demonstrated 60.6% sensitivity and 100% specificity for nested PCR targeting the 100‐kDa‐like‐protein gene in 33 histopathologically proven histoplasmosis FFPE cases.^31^ For PCR of the 28S region, Willinger et al.^18^ reported an 87% sensitivity. In another comparative study, Rickerts et al.^32^ showed acceptable sensitivity for molecular diagnosis of invasive aspergillosis and mucormycosis. Lau et al. reported 82% positive results for panfungal PCR assay.^33^ They also showed 97% and 68% sensitivity for fresh and FFPE samples, which was lower than the current study results. Cabaret et al. indicated 62.5% and 93.75 positive results of FFPE samples for conventional PCR and qPCR, respectively.^34^ These differences may result from the differences in amplicon length (>300 bp for conventional PCR vs. 150 bp for qPCR) and the type of PCR method. Hammond et al. reported 81.5% positive results in a similar study by semi‐nested PCR of 18S rDNA using ZM1/ZM2 and ZM1/ZM3 primer pairs targeting an amplicon <200 bp.^35^ Salehi et al. reported a 64% sensitivity for identifying fungi from FFPE by qPCR assay.^21^ Drogari‐Apiranthitou et al.^36^ reached 45% positive PCR for *Mucorales* and 40% positive for *Aspergillus* spp. They reported 79.3% sensitivity and 100% and specificity for semi‐nested PCR, respectively. The higher sensitivity of their method compared to our study might be due to the thickness of their tissue cuts (10 μm vs. 5 μm). In another investigation, Ganesan et al. found that the location of infection may alter the sensitivity of the PCR test, with sensitivity increasing from 63% to 83.3% for angioinvasion sites.^16^ Although the site of the infection may affect the results, to set up a method independent of histopathological tests, in this study, a part of the sample was randomly taken from a PEB. While Jung et al. reported a 41.3% positive rate for panfungal PCR due to long amplicon length and perhaps an ineffective DNA extraction kit.^37^ This work found a concordance rate of 76% between semi‐nested PCR of the ITS region and histopathology tests, while others reported higher^21^ or lower^38^, ^39^ concordance rates between these two methods.

Due to the small amount of tissue in FFPE samples, the extracted DNA would be low. Hence, the selection of a proper extraction method is a key step in achieving reliable amplification. We used QIAamp DNA FFPE Tissue Kit (QIAGEN), as successfully used before.^18^, ^19^, ^36^, ^38^, ^40^, ^41^, ^42^, ^43^, ^44^ In a comparative study between commercial extraction kits, Muñoz‐Cadavid et al. reported that TaKaRa and QIAamp extraction kits yielded the best results for extracting high‐quality DNA from the FFPE sample.^45^

Similar to some previous studies,^21^, ^27^, ^35^, ^36^, ^43^, ^45^ we used the human β‐globin gene as internal control, and the primers successfully amplified the expected region in all samples, while others used different genes, such as IRBP,^39^ GAPDH,^31^ and ZP3,^46^ as controls.

In this work, we used a panfungal semi‐nested PCR to identify fungal elements in FFPE tissue samples by amplifying the ITS1‐5.8s‐ITS2 followed by ITS1 regions, which is consistent with a prior result.^47^ Several researchers amplified other targets for detecting fungal DNA in FFPE samples, including 28S rDNA,^19^ ITS2,^16^ 18S rDNA,^36^, ^37^ and mitochondrial tRNA.^36^ Even though Cabaret et al. claim that targeting mitochondrial DNA is preferable to ITS,^34^ they got a lower positive rate by conventional PCR (10/16, 62.5%) than we did (56/64, 87.5%). In another study, Jillwin et al.^20^ targeted five gene regions, including universal ITS (ITS1‐5.8s‐ITS2), ITS1, ITS2, 18S rDNA, and D1/D2 of 28S rDNA, and reported that ITS1 amplification led to 61.9% positive results by the PCR method.

The following factors may contribute to false‐negative or false‐positive outcomes: first, artifacts during staining cause false‐positive histopathology findings. Second, the presence of conserved genes in multiple copies (rDNA) is a disadvantage in clinical specimens collected from nonsterile body sites because nonpathogenic commensal fungi, environmental spores, or colonizing fungi can also cause significant nonspecific amplification in samples primarily composed of human cells with a few fungal cells. Third, the formation of protein‐DNA cross‐links and fragmentation of DNA during the fixation process result in a lack of intact DNA required for amplification. More specifically, it is difficult to amplify the target gene when DNA is highly fragmented or cross‐linked and has a large amplicon size. Fourth, the presence of amplifiable fungal DNA in tissue does not always imply the presence of a housekeeping gene (human‐globin).

As *Aspergillus* species and Mucorales are the most frequent causative agents of FRS, it can be a good idea to use species‐specific primers and multiplex PCR to differentiate them as soon as possible in a single reaction. To achieve greater sensitivity and specificity rates for PCR findings, it is advised that future research uses a shorter amplicon length^30^ and execute both procedures in a single tube to prevent contamination.

When compared to histopathologic and microbiological approaches, molecular diagnostics offer both advantages and limitations. FRS was found and identified in this work by panfungal primers and subsequent sequencing. Panfungal PCR techniques benefit from detecting any fungal DNA, even that of uncultured, unusual, or unfamiliar fungi. Generally, panfungal PCR techniques are used in conjunction with Sanger sequencing of amplicons, requiring single‐species PCR results and increasing the time to diagnosis. Costs and lack of standardization are two of the key drawbacks to PCR‐based techniques. Furthermore, the kind and quality of sample material may have an impact on the results. As a result, further analysis is still required for technological improvements and enhancements in these molecular tests.

## 5 CONCLUSION

In this study, semi‐nested PCR amplification of ITS1‐5.8S‐ITS2 in IFI FFPE samples yielded a significant result with increased sensitivity and specificity. Given the small number of samples obtained during endoscopy‐guided biopsy and the rapid clinical progression of rhinocerebral mucormycosis, establishing a fast, precise, and reliable molecular technique for direct detection of FRS in FFPE samples may improve results. In this paper, we propose that semi‐nested PCR might be a reliable supplemental tool for histology experiments.

## CONFLICT OF INTEREST

The authors declare that they have no competing interests.

## AUTHOR’ CONTRIBUTIONS

**Mohammad Javad Ashraf and Kayvan Pakshir** validated the study, conceptualized the study, and contributed to methodology. **Mohammad Kord and Foroogh Shamsizadeh** investigated the study, involved in formal analysis, and wrote—review & editing. **Hamid Morovati** wrote—original draft and wrote—review & editing. **Saham Ansari involved in** formal analysis and contributed to methodology. **Golsa Shekarkhar and Hamid Badali** wrote—review & editing. **Bijan Khademi and Mahmood Shishegar** curated the data and provide resources. **Kazem Ahmadikia provided s**oftware, investigated the study, and involved in formal analysis. **Kamiar Zomorodian** validated the study, supervised the study, involved in project administration and finding acquisition, and wrote—review & editing.

## Data Availability

The thirty‐five sequences generated in this study were deposited in the GenBank database (accession numbers from MZ333236 to MZ333270 with persistent accessible links from MZ333236 to MZ333270, respectively). All data used to support the findings of this study are available from the corresponding author upon request.
